# Community trust, not resource pledges alone, will determine whether the DRC contains its seventeenth Ebola outbreak

**DOI:** 10.1016/j.nmni.2026.101832

**Published:** 2026-08-25

**Authors:** Abdullahi Abdisalam Mohamed, Mohamed Mustaf Ahmed

**Affiliations:** a Department of Public Health, Faculty of Medicine and Health Sciences, SIMAD University, Mogadishu, Somalia; b Faculty of Medicine and Health Sciences, SIMAD University, Mogadishu, Somalia; c School of Global Health, Chulalongkorn University, Bangkok, Thailand

Dear editor,

The Ebola outbreak that began in Ituri Province in May 2026 became the seventeenth recorded in the Democratic Republic of Congo, and the joint call issued by the World Health Organization and the Africa Centres for Disease Control and Prevention on August 6 for an urgent scale-up of community-led response deserves close attention rather than routine endorsement [1]. As of August 4, the country had reported 3973 confirmed cases, 1801 deaths, and 776 recoveries across 51 health zones in five provinces, with 99 new cases and 52 deaths recorded in a single 24 hour period [1].

This commentary argues that the technical measures the call rightly emphasizes, early symptom recognition, contact follow up, infection prevention in health facilities and support for frontline workers, will fail to translate into containment unless they are paired with a deliberate and adequately resourced strategy for rebuilding community trust in a region where that trust has been repeatedly damaged.

Uganda's experience offers a useful point of comparison rather than a template that can be transferred wholesale. Following its outbreak declaration, Uganda recorded only 20 confirmed cases and two deaths before declaring the end of transmission on July 28 a result attributed in part to early detection, coordinated national action, and trusted community engagement [2]. The scale, insecurity, and institutional distrust that characterize the current DRC outbreak differ substantially from the Ugandan context, and this difference appears to explain much of the divergence in outcomes [3].

The contact tracing figures reported alongside the August 6 call illustrate the practical cost of this deficit in trust. Only 75 percent of known contacts in the DRC are currently being followed up, well below the 95 percent target considered necessary to interrupt transmission chains quickly [1]. A shortfall of this magnitude is rarely attributable to logistical capacity alone, and evidence from the 2018 to 2020 DRC outbreak suggests that a heavily biomedical approach that sidelines community grievances tends to generate resistance that undermines this kind of follow-up work [4]. More than 450 acts of violence or threats against health workers were documented during that earlier epidemic, and the response only began to gain traction once strategic plans were revised to place community engagement, logistics, and security on an equal footing with clinical measures [5].

Community-based surveillance systems developed during that same period demonstrated that early and consistent community involvement can strengthen both disease detection and access to care, and that this involvement was a critical factor in local acceptance of response activities [6]. A separate review of the humanitarian approach taken in the DRC reached a more critical conclusion, questioning how far affected communities were genuinely brought into decision-making rather than simply consulted after national and international actors had already set the direction of the response [4]. Commentators have also observed that what looked to outside observers like irrational resistance to Ebola control measures often reflected a coherent response to decades of conflict, marginalization, and prior encounters with external actors, a distinction that carries direct implications for how the current call for community-led action should be operationalized [7].

Treatment center occupancy in North Kivu has reportedly reached 139 percent of capacity, a figure that places acute pressure on health workers and risks further eroding confidence in the system precisely when that confidence is most needed [1]. Expanding bed capacity and clinical supplies is necessary, but it is unlikely to be sufficient if patients and their families continue to associate treatment centres with fear rather than care. The lesson from earlier outbreaks is that trust is not a byproduct of an effective response but a precondition for one, and that resourcing decisions should be evaluated in part by whether they visibly address the everyday concerns of affected communities rather than only the epidemiological indicators that responders track.

This study has three implications. First, the 95 percent contact follow-up target should be treated as a diagnostic of community relations as much as of operational capacity, and persistent shortfalls should trigger a review of engagement practices in the affected health zones rather than only additional staffing. Second, resources directed toward frontline health workers should be paired with visible, near-term benefits to host communities, following the precedent set by cash-for-work and infrastructure programs that appeared to improve acceptance during the 2018 to 2020 outbreak [5]. Third, evaluations of the current response, including those conducted jointly by the WHO and Africa CDC, should report indicators of community trust and engagement alongside case counts and mortality figures so that the balance between biomedical and social investment can be assessed transparently as the outbreak evolves [6]. Without such indicators, calls for community-led action risk remaining rhetorical, even as they are repeated with each new outbreak.

## CRediT authorship contribution statement

**Abdullahi Abdisalam Mohamed:** Writing – original draft. **Mohamed Mustaf Ahmed:** Writing – review & editing.

## Ethical approval

Not applicable.

## Funding

The authors did not receive any funding for this study.

## Declaration of competing interest

The authors declare no competing interests.
